# Darmok and Jalad at the Psych Ward: A Case Demonstration of How to Creatively Communicate with a 19-Year-Old Patient with Autism Spectrum Disorder

**DOI:** 10.1155/2021/6690564

**Published:** 2021-02-24

**Authors:** Elizabeth Kim, Katherine Martin, Laurence Karper, Siddhartha Maru, Maggie Driscoll

**Affiliations:** Lehigh Valley Hospital-Muhlenberg, Bethlehem, PA, USA

## Abstract

Difficulties in communication often arise between individuals with autism spectrum disorder and their treating physicians because both sides struggle to find a common ground. The story of Darmok and Jalad at Tanagra from Star Trek: The Next Generation nicely exemplifies how two populations that spoke different languages were still able to find a creative way to communicate with each other. This story is used as a metaphor to illustrate how a novel connection was made with a 19-year-old patient with autism spectrum disorder who was admitted to the inpatient psychiatric unit.

## 1. Introduction

Patients with autism spectrum disorder have proven to be some of the most challenging populations to work with on inpatient psychiatric units. Difficulties arise between these patients and their treating physicians because both sides struggle to find a common ground ([1, 2]). Challenges in verbal communication skills, need for consistency, lack of reciprocity, and difficulty understanding theory of mind hinder these patients' ability to connect with their physician. For two-way communication to be effective, physicians also have a responsibility to meet these patients at their level. To accomplish this goal, physicians must make accommodations to facilitate reciprocity, identify patients' interests, and utilize those interests to ease communication [3]. The need arises for physicians to think outside of their own communicative methods and to learn those of their patients. The story of Darmok and Jalad at Tanagra from Star Trek: The Next Generation [4] nicely exemplifies how two populations that spoke different languages were able to find a creative way of communication. This story was used as a metaphor to help illustrate how an inpatient psychiatric treatment team was able to effectively communicate with a 19-year-old patient with autism spectrum disorder.

In the story of Darmok and Jalad, the Federation attempts to communicate with the Tamarians to establish diplomatic relations. However, the Tamarians speak a language incomprehensible to their universal translator who is supposed to interpret the aliens' language for the crew. In anticipation of the language barrier between the two populations, Picard from the Federation astutely points out that “communication is a matter of patience, imagination… these are qualities for which we have insufficient measure.” Picard and Dathon, a Tamarian, are transported from their respective ships to the surface of El-Adrel, where they initially struggle to communicate with each other. Dathon repeatedly remarks, “Darmok and Jalad at Tanagra,” which initially confuses Picard. Abiding by his words and using patience and imagination, Picard begins to comprehend Dathon's speech and eventually realizes, “that's how (the Tamarians) communicate, by citing example, by metaphor.” He begins to comprehend that “Darmok and Jalad at Tanagra” was an event that exemplified how a danger shared between two people can bring the two people together and that Dathon was applying that example to their current situation of Dathon and Picard here at El-Adrel. At that point, Picard has grasped Dathon's language enough to converse with him, the language once deemed incomprehensible.

The fictional case from Star Trek has rather practical implications for communication in clinical settings. Patience and imagination proved to be invaluable tools and the necessary measures to establishing a therapeutic alliance and effective communication with the patient in our case.

## 2. Case Presentation

The patient was a 19-year-old African American male with autism spectrum disorder and schizoaffective disorder. He was initially admitted for bizarre behavior, grandiose delusions, erotomania, depression, and suicidal ideation. He expressed grandiosity of saving the universe and delusions about being in a relationship with a female peer whom he had met in school and considered to be his “girlfriend.” He was stabilized on olanzapine for psychosis and fluoxetine for obsessional thoughts. His treatment team worked with him to let him know that he could think about this female peer but that he should not call or visit her. The patient agreed, albeit reluctantly. He was discharged once he was no longer suicidal and his psychosis had abated.

Within seven days of discharge, he was readmitted with continued erotomania and plans to visit this female peer. He also expressed some homicidal ideation towards his family for preventing him from seeing her. He was sexually preoccupied during the admission and acted inappropriately around female patients. Collateral information from his sister revealed that being discharged, and once again being near his “girlfriend's” home, may have triggered him. She noted that he had been compliant with his medications, so medication noncompliance was not a cause for his decompensation. Nevertheless, his medications were readjusted. He was cross-tapered from olanzapine to haloperidol and then to aripiprazole. Fluoxetine was discontinued in case it was having unwanted activating side effects. Medications proved beneficial for the patient's overall psychotic symptoms but did not address his inappropriate behaviors on the unit. However, a novel way of communication managed to fill that gap.

The patient frequently verbalized that an asteroid was going to hit the earth and that we would all die from the asteroid. He often expressed grandiose thoughts of needing to save the world from the asteroid. He loved to draw, and many of his drawings depicted an asteroid shooting towards the earth. Over several weeks, the treatment team expressed curiosity about his drawings and encouraged discussion about them. Eventually, he had enough spontaneity to discuss the past trauma of his mother's partner abusing him and his sisters. The treatment team then made the connection that his mother's partner, like the asteroid about to hit the earth, was an example of how one bad thing could make everyone suffer. The patient, who had never expressed much interest in the team's responses to him, exclaimed “Yeahhhh!” as he widened his eyes and smiled. He also offered a fist pump, which was the first time he initiated reciprocal communication with the team. With several weeks of patience and imagination, the treatment team began to learn how to speak to him on his own terms.

Patience and imagination also proved to be essential in addressing his inappropriate behaviors on the unit. The team had learned from his prior admission and previous attempts to redirect his behavior, that directly telling him not to engage in certain behaviors (e.g., keeping his hands to himself and not using offensive language) was rather futile. During the second admission, the team decided to take a different approach. It was apparent that the patient's drawings were a way of communication for him. While he typically responded with brief one- or two-word answers to routine psychiatric interview questions, he went into lengthy descriptions about his drawings when prompted. His drawings became a way for him to express his thoughts and emotions and to facilitate discussion with the treatment team.

Inspired by the patient's prior drawings, the team drew the Earth with a protective shield made of stick figures representing people on the unit and an asteroid coming towards the earth. The patient, who had previously exhibited distractibility and poor eye contact, suddenly paid very close attention to the picture being drawn. The team explained to him that the patient was a part of the protective shield and pointed out one of the stick figures to represent him. He was told that he was an important part of the shield and by maintaining appropriate boundaries along with everyone else who was a part of the shield, he could make others feel safe and prevent the asteroid from hitting the Earth. The patient expressed understanding and did not oppose. While his initial response was not totally affirmative, it was a more positive outcome than in the past when he had been given verbal instruction alone.

Several weeks after he was encouraged to engage in appropriate behavior by using pictures, the team was informed by a group therapy facilitator that the patient had said, “we need to be kind to everyone on this earth… so everyone will survive.” Not only did the patient understand the message the team was conveying at the time the picture was drawn but he also remembered and carried that message with him several weeks out. Incorporating pictorial communication with verbal communication was a way the team successfully modified its ways to meet his needs and to make communication more effective. Ultimately, he was able to be stabilized with no further instances of concerning behavior to a group home.

## 3. Discussion

This case parallels the story of Picard and Dathon in Star Trek. As stated by Picard, patience and imagination were virtues that enabled communication with those who speak a language different from their own. As Gernsbacher points out, “Successful healthcare interactions depend on provider's reciprocity, that is, their willingness and ability to modify their own behavior to meet patient needs and treat them with respect” [5] when working with autistic patients. The term reciprocity implies effort from both the patient and the physician. When the patient has barriers preventing them from fulfilling their end, the physician must go one step further to meet the patient where they are at. In the case described above, the patient exhibited reciprocal communication including expressive response, eye contact, and fist pump after the team took one step further to make a connection using a metaphor revolving around his interests.

Physicians' willingness to provide accommodations has shown to significantly impact the success of patient-physician interactions [6], and this case exemplifies how several modifications in communication facilitated the interactions—conveying understanding of the patient's life experiences by rephrasing it with his metaphors, recognizing that his expressions and drawings may seem repetitive and bizarre but carry deeper meaning once explored, understanding that his drawings offered additional insight to his thoughts, building rapport by expressing curiosity about his drawings, and offering alternative communication methods. While there are many well-known ways to accommodate communication with autistic patients such as simplifying sentences, reducing sensory stimulation, and communicating by writing, this case demonstrates some novel methods that required thinking outside the box on the physician's end.

## 4. Conclusions

In summary, individuals with autism spectrum disorder can be challenging to communicate with in clinical settings. Thinking outside the box and employing creative strategies to connect with such patients can be highly effective. Using the story of Darmok and Jalad at Tanagra from Star Trek: The Next Generation as a metaphor, the treatment team was able to very effectively connect to and communicate with a 19-year-old male with autism spectrum disorder. In general, this case highlights that certain populations of patients may require accommodations based on their individual life experiences, talents, and topics of interest.
